# Supplementary material for: Earliest long-necked sauropterygian Lijiangosaurus yongshengensis and plasticity of vertebral evolution in sauropterygian marine reptiles
Source: Commun Biol. 2025 Nov 11;8:1551. doi: 10.1038/s42003-025-08911-1 (PMC12606328; doi:10.1038/s42003-025-08911-1)
Supplement: Supplementary file 1 — Supplementary Information [file 42003_2025_8911_MOESM1_ESM.pdf]

Supplementary Information

Earliest Long-necked Sauropterygian *Lijiangosaurus yongshengensis* and Plasticity of Vertebral Evolution in Sauropterygian Marine Reptiles

Wei Wang[1], Qinghua Shang[1]*, Jiansheng Wang[2], Hongke Zi[3], Chun Li[1]

1 Institute of Vertebrate Paleontology and Paleoanthropology, Chinese Academy of Sciences, 100044 Beijing, China.
2 Yunnan Biantun Cultural Museum of Yongsheng, 674200 Lijiang, Yunnan, China.
3 Lijiang Municipal Administration of Culture and Tourism, 674100 Lijiang, Yunnan, China
* Corresponding author: shangqinghua@ivpp.ac.cn

Additional description

General preservation.

The skull and the mandible are articulated though slightly displaced from the atlas. All the cervical, the anterior dorsal, and the middle series of caudal vertebrae are preserved in articulation. Although the posterior dorsal, the sacral, and the proximal and distal caudal vertebrae are missing possibly due to weathering, according to the posture of this skeleton, we can conclude that the other preserved bones of the vertebral column, the pectoral and pelvic girdles, and the four limbs mostly remain in situ.

The skull is measured 18.2 cm from the anterior extreme of the dentary to the occipital condyle along the midline. The preserved regions of the axial skeleton are directly measured including a complete neck of 115 cm, and we speculate that the trunk is about 80 cm and the tail is no less than 40 cm long. Hence, the skull is relatively small, less than 8% of the total length of the animal.

Skull.

The skull was severely damaged owing to prolonged exposure to weathering. It is exposed in ventral view, and it is slender, gracile, and generally triangular, with the lower jaw preserved in articulation (Fig. S4). The oval right internal naris or choana is partially visible, and its posterior margin is at the level of the suture between the premaxilla and the maxilla, which indicates a well-developed snout formed by an elongate premaxilla. Despite being crushed with an obscure anterior part, the paired vomers can be identified separating the internal nares. Posteriorly, the vomer extends far beyond the level of the posterior margin of the internal naris, and sutures to the anterior process of the pterygoid. Only the anterior part of the right palatine is preserved, and its relatively narrow anterior end defines the posterior margin of the internal naris. There may be a choanal groove on the anterior end of the palatine. The palatine anteromedially meets the vomer. The ventral surface of the pterygoids is deeply

depressed along the midline suture, forming a wide trough posteriorly. The anterior and posterior regions of the pterygoids are preserved, and the outline of the nearly entire pterygoid can be defined. The quadrate rami of the pterygoids subject to extensive breakage, but sufficient portions are preserved to demostrate their relatively straight morphology and posterolateral divergence respectively connecting to the quadrates.

Medial to the posterior shaft of the quadrate, there is an opening of the cranio-quadrate passage (Fig. S4). It can be seen that the matrix filled inside the passage due to the damage of the mandibular condyle of the quadrate forming the ventral part of the passage. The cranio-quadrate passage opening is defined by the pterygoid medially, the shaft of the quadrate laterally, and the occipital flange of the squamosal dorsally.

Besides the occipital condyle, the other part of the basioccipital is narrowly exposed between the posterior edges of the pterygoids and the occipital condyle. The occipital condyle points backward with the horizontal width of 10 mm and the vertical height of 7 mm. Lateral to the occipital condyle, the basioccipital carries a pair of basioccipital tubers with horizontal lengths of 6 mm and vertical diameters of 6 mm.

Lower jaw.

The anterior half of the lower jaw, mainly including the dentaries and possibly the splenials, is preserved in articulation with the upper jaw (Fig. S4). There is no distinct constriction at the lateral margins of the rostrum. The mandibular symphysis is moderately developed with the maximal length of 33 mm and the maximal width of 28 mm, and this results in a length-to-width ratio of 1.18, which is far less than the species of *Nothosaurus* and slightly more than the species of *Lariosaurus*, when the corresponding ratio is 1.5-1.7 in *Nothosaurus mirabilis*, 1.47 in *Nothosaurus jajisteus*, 1.13 in *Lariosaurus calcagnii*, and 0.9-1.0 in *Lariosaurus buzzii*.

Dentition.

YSBB208 shows the relation of the upper and lower dentition relative to one another with the jaws closed (Fig. S3). Most of the teeth possess evident striae on the lateral surfaces of the crowns. The premaxilla fangs or tusks are present, although the exact numbers are unknown. There are at least three premaxillary fangs with cross sections remaining, and they respectively fit in between the anterior dentary fangs. There is one distinctly enlarged fang on the maxilla compared to other maxillary teeth. Possibly four small conical teeth set anterior to the maxillary fang, and more than nine small conical teeth are developed posterior to it. Although the posterior end of the maxilla and also the ectopterygoid are not preserved, according to their impressions, the maxillary tooth row possibly extends backward to a level beyond the posterior corner of the orbit and even the anterior corner of the upper temporal fossa. Three elongate dentary fangs are

clearly retained, and two more large alveoli are observed from the fine convex ridges on the surface of the left dentary, and therefore, the rostral region of the dentary bears five fangs. A series of small conical teeth posterior to the last fang on the dentary with residual occlusion of the maxillary teeth.

Vertebral column.

The most anteriorly preserved vertebral element is the atlas centrum (Fig. S5 and S6), though slightly damaged, is shaped very differently and much smaller compared with the axis centrum and other cervical centra. Its anterior articular surface is a somewhat spherical structure, and the posterior part is a short cylindrical object. No atlas intercentrum or atlas ribs are found. A small bump on the lateral surface near the anterior part represents the atlas rib facet. The axis centrum is much larger than the atlas and has the same morphology as the following vertebrae, which develop cylindrical and laterally constricted centra. The ventral surface of the axis bears an evident longitudinal keel. There is a split, broken only on the ventral surface but absent in the dorsal portion, at the anterior portion of the axis centrum forming a disk-like object. Based on its serrated fractures, we consider this object as part of the axis centrum rather than the axis intercentrum. In the axis centrum, a bump facet on the lateral surface near the anterior end represents a broken axial rib.

The anterior cervical centra shows a strong lateral constriction. Progressing backward, the lateral constriction becomes gradually reduced to moderate. The articular surface of the centrum is platycoelous. From the axis to the 34th cervical, there are a pair of longitudinal shallow concavities divided by a midline keel on the ventral surface of the centrum (Fig. S5 and S6). Progressing backward within the cervical series, the ventral surface of the centra gradually becomes smooth. As typical in sauropterygians, the cervical rib is bicipital with a free anterior process in this new skeleton. In the anterior part of the cervical region from the axis to about the 30th cervical vertebra, the articular heads of the rib attach to the diapophysis and parapophysis respectively that are represented by two horizontally elongated oval pits both located on the centrum (Fig. S5 and S6). Processing backward from the 31st to the 37th cervical vertebra, the neural arch participates in the formation of the diapophysis, which is consequently traversed by the neurocentral suture, whereas the parapophysis continues to be formed by the centrum only. From the 38th to the 42nd cervical vertebrae, the parapophysis either fuses with diapophysis and transfers to the neural arch or disappears gradually (Fig. S5 and S6). The cervical ribs gradually increase in length from the front to the back. The anterior cervical ribs, such as the 5th cervical, possess anterior processes that are nearly as long as the posterior processes. From the middle to the posterior cervical regions, the posterior process becomes longer, while the anterior process becomes shorter to absent

from about the 30th cervical rib.

The centra of dorsal vertebrae are short cylinders showing smooth ventral surfaces and weakly constricted bodies. Some scattered dorsal centra expose their articular surfaces, which are platycoelous or attain a little amphiplatyan condition. The transverse process of the dorsal vertebra is short and stout that projects laterally from the neural arch, and the articular surface to the rib is oblong with the height twice the width. Most of the neural spines of the preserved dorsal vertebrae are not exposed resulting in uncertain morphology. Only the 6th to 8th dorsal neural spines, which are folded and incomplete, can be observed from the side of the rock block. The 6th and 7th dorsal neural spines are wider than tall, and each shows a middle constriction and dorsal expansion in anteroposterior orientation. In contrast, the height of the 8th dorsal neural spine is twice its width (Fig. S5 and S6).

In the articulated caudal vertebral series, their neural spines are somewhat posterodorsally inclined and rectangular with the heights slightly longer than their widths. The dorsal terminal of the caudal neural spines is expanded both at its anterior and posterior terminals resulting in a compression in between. There are about eight caudal centra scattered anterior to the femurs and about four additional ones posterior to them. These centra possibly from the distal caudal region are amphiplatyan and laterally constricted with the length longer than the width, and develop small round articular faces to caudal ribs. In dorsal view of an isolated caudal vertebra dislocated to be preserved near the knee, the sutural facet receiving the pedicel of the neural arch expands into a cruciform platform (Fig. S5 and S6). A few bar-like and distally pointed disarticulated caudal ribs or chevrons are identified.

Gastralia.

Several elements of disarticulated gastralia are displaced in the trunk region. The medial element is in boomerang shape and develops a distinct anterior process, and its lateral shanks enclose a wide angle of more than 100° (Fig. 1).

Pectoral girdle and forelimb.

In the pectoral girdle, the clavicles, the coracoids, and the right scapula are preserved though disarticulated (Fig. 1 and S7). Both the pair of clavicles and the left coracoid are displaced, whereas the right coracoid, scapula, and humerus remain in situ and exposed in ventral view. Neither coracoid is completely exposed, but we can readily identify the entire outline because these two coracoids complement each other well (Fig. 1).

A ventral concavity on the proximal truncated end of the clavicle that accepts the lateral

process of the interclavicle can be defined. The tapering posterolateral process of the clavicle possibly applies to the medial portion of the scapula as a typical pattern of sauropterygians. The platelike bone overlapped by the possible left clavicle and exposed as a triangle might be the interclavicle, and its morphology is unknown except for a plausible laterally projecting process.

The scapula is again of a typically nothosaurian structure, although only the ventral glenoidal portion is well preserved and the posterodorsally ascending process or blade is unknown. On this glenoidal portion, the concave anterior edge is slightly weathered but appears to approximate the original edge, and its medial margin is concave. According to the broken dorsal part of the scapula, a blade is possibly well-developed.

The ulna and radius were displaced and inverted before burial. The radius is more slender than the ulna, and they are both expanded at the ends with constricted shafts. The preaxial margin of the radius is relatively straight and its postaxial margin is concave. No carpal was preserved, and only the possible first and second metacarpals remained.

Pelvic girdle and hind limb.

The elements of the pelvic girdle remain in situ and articulate with the right hindlimb, but only the incomplete right pubis and two fragments from the right ischium and ilium are preserved (Fig. 1 and S8). The acetabular portion of the ischium shows a rounded and convex proximal end. The ilium is small with only its medial concaved acetabular portion exposed, whereas the iliac dorsal process is not complete.

Both the right and the left hind limbs are preserved, when the right side is in better condition. The right femur is complete and only slightly longer than the humerus. The femoral shaft is slender and weakly sigmoidally curved. The internal trochanter on the femoral head is reduced, and the intertrochantic fossa on the femoral distal end is rudimentary. The tibia and the fibula are almost equal in length both without distinct constriction, whereas the fibula is more concave at its proximally axial margin and more slender than the tibia. In the left hind limb, the orientation of the concave lateral axial margin of the fibula indicates its displacement and perversion before burial.

There are only two roughly rounded tarsal ossifications, the astragalus and the calcaneum. The concave proximal rim of the astragalus attaches to the distal ends of both the tibia and the fibula. The calcaneum attaches to the laterodistal end of the fibula and is smaller than the astragalus. The right and the left first metatarsals are both completely preserved, which are proximally expanded and much more stout than other metatarsals. The possible right metatarsals II and III and the potential left metatarsal V are rather straight, slim, and obviously longer than the first metatarsals.

Supplementary figures

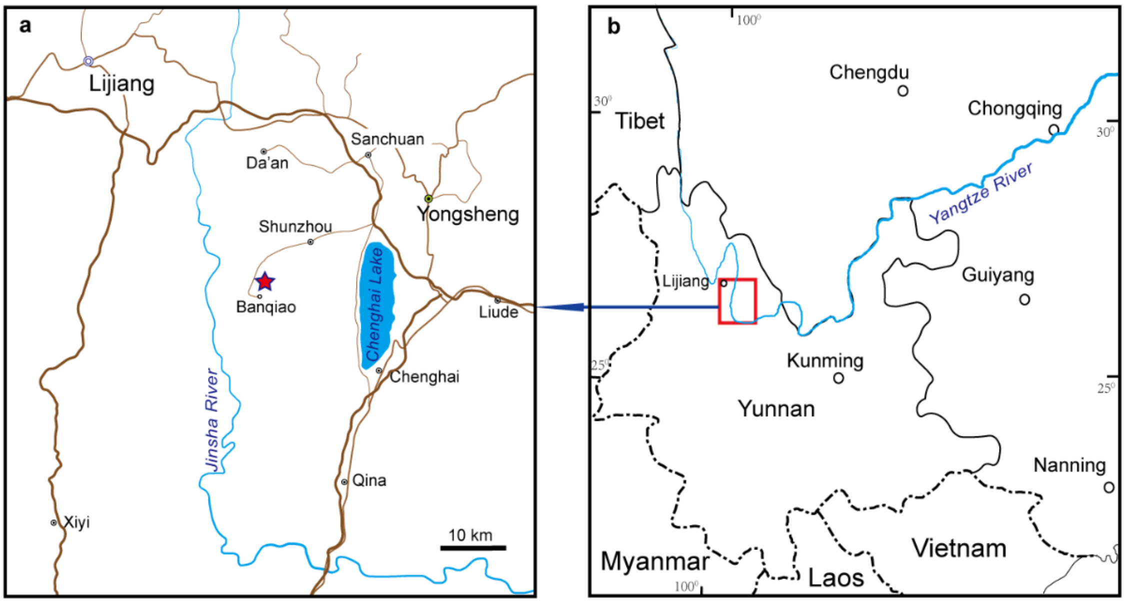

Figure S1. Map showing the fossil locality of *Lijiangosaurus yongshengensis*. The type specimen reported in this study was collected from the site (marked with a star) near Yongsheng town of Lijiang city (a) in the northwestern region of Yunnan province, southwestern China (b).

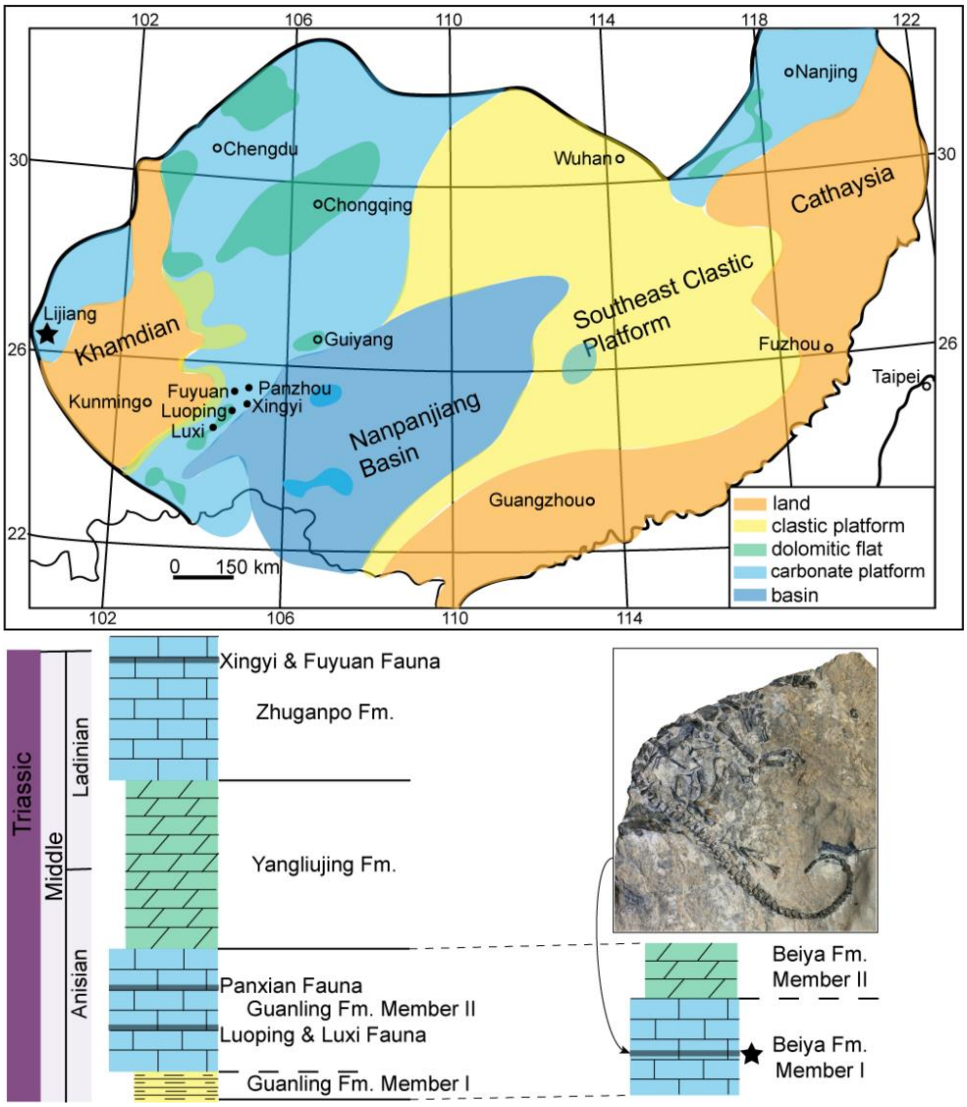

Fig. S2 Geological map showing Triassic marine reptile fossil localities and strata in southwestern China. Most of the fossil sites (black dots) are in the east of the Khamdian Oldland, whereas the new site in Lijiang is in the west (black star), and the base map is after Feng et al (2017). The Beiya Formation is comparable in age to Member II of the Guanling Formation of early Anisian, the early Middle Triassic.

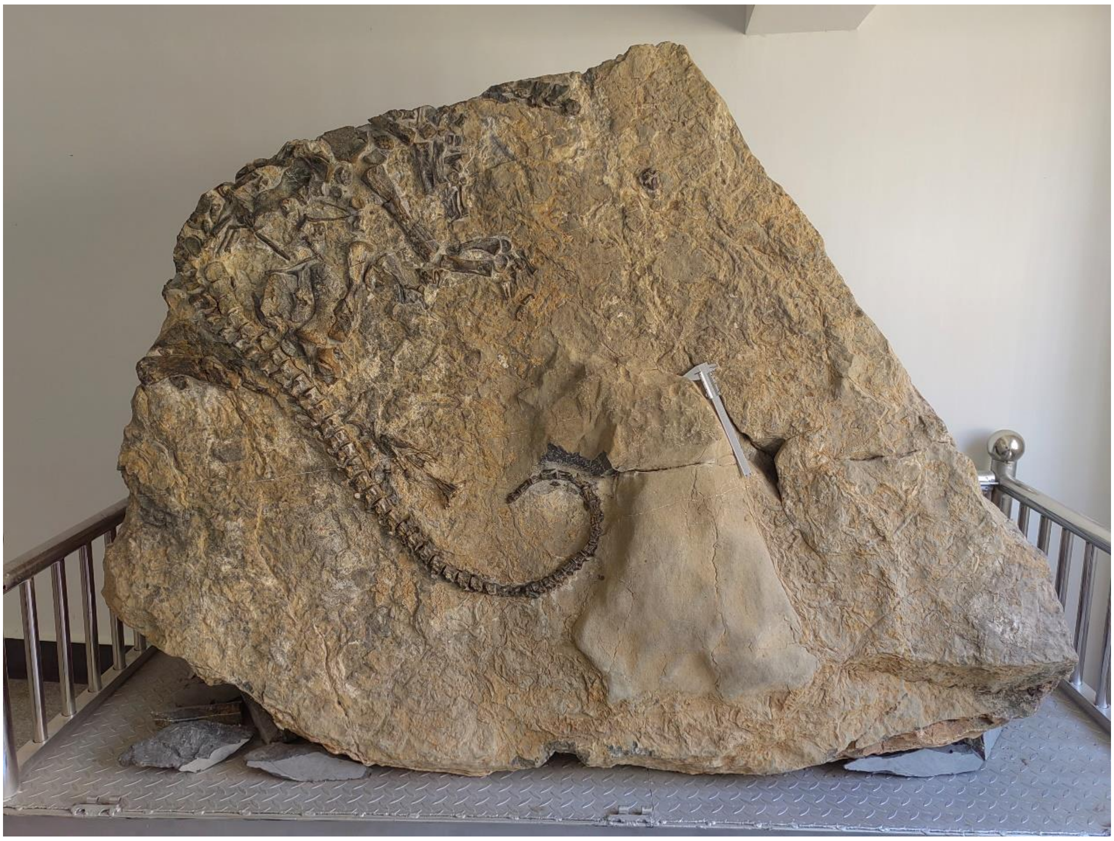

Fig. S3 The large limestone block with the incomplete skeleton of the holotype of *Lijiangosaurus yongshengensis* (YSBB208) preserved.

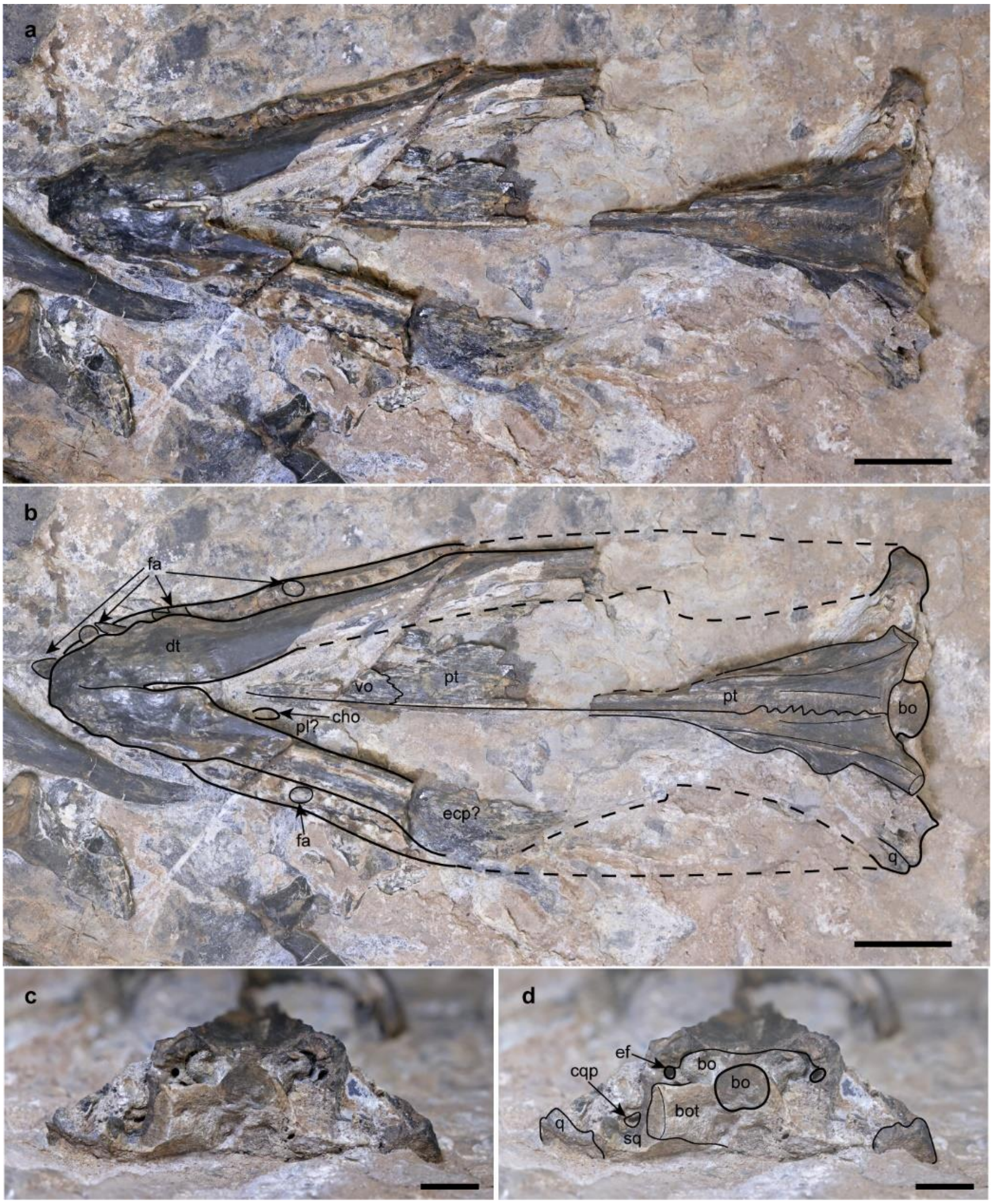

Figure S4. Skull of the holotype of *Lijiangosaurus yongshengensis* (YSBB208). a, image of the skull in ventral view; b, osteological interpretation of the skull in ventral view; c, image of the occiput of the skull in posterior view; d, osteological interpretation of the occiput. Abbreviations: bo, basioccipital; bot, basioccipital tuber; cho, choana; cqp, cranio-quadrate passage opening; dt, dentary; ecp, ectopterygoid; ef, eustachian foramen; fa, fang; pl, palatine; pt, pterygoid; q, quadrate; sq, squamosal; vo, vomer. Scale bars are 2 cm in a and b, 1 cm in c and d.

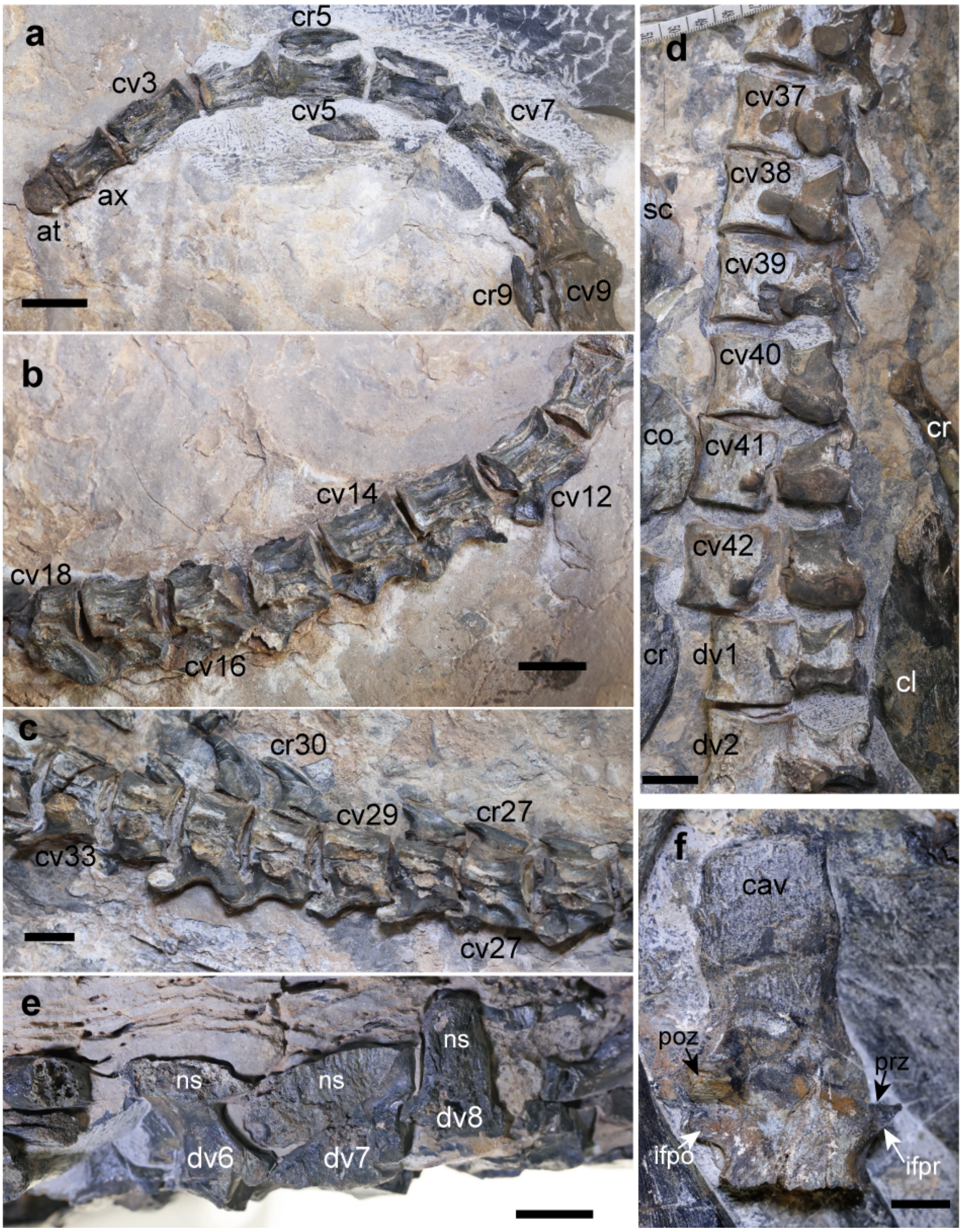

Figure S5. Cervical and anterior dorsal vertebrae of the holotype of *Lijiangosaurus yongshengensis* (YSBB208). a, the first to 9th cervical vertebrae in ventral view; b, the 11th to 18th cervical vertebrae in ventral view; c, the 26th to 33rd cervical vertebrae in ventral view; d, the 37th to 42nd cervical and the most anterior two dorsal vertebrae in left lateral view preserved near the pectoral girdle; e, the neural spines of the 6th to 8th dorsal vertebrae in left lateral view exposed on the side of the stone block; f, an isolated anterior caudal vertebra in right lateral view. abbreviations: at, atlas; ax, axis; cav, caudal vertebra; cl, clavicle; co, coracoid; cr, cervical rib; cv, cervical vertebra; dv, dorsal vertebra; ifpo, infrapostzygapophysis; ifpr, infraprezygapophysis; ns, neural spine; poz, postzygapophysis; prez, prezygapophysis; sc, scapula. Scale bars are 1 cm in f and 2 cm in other images.

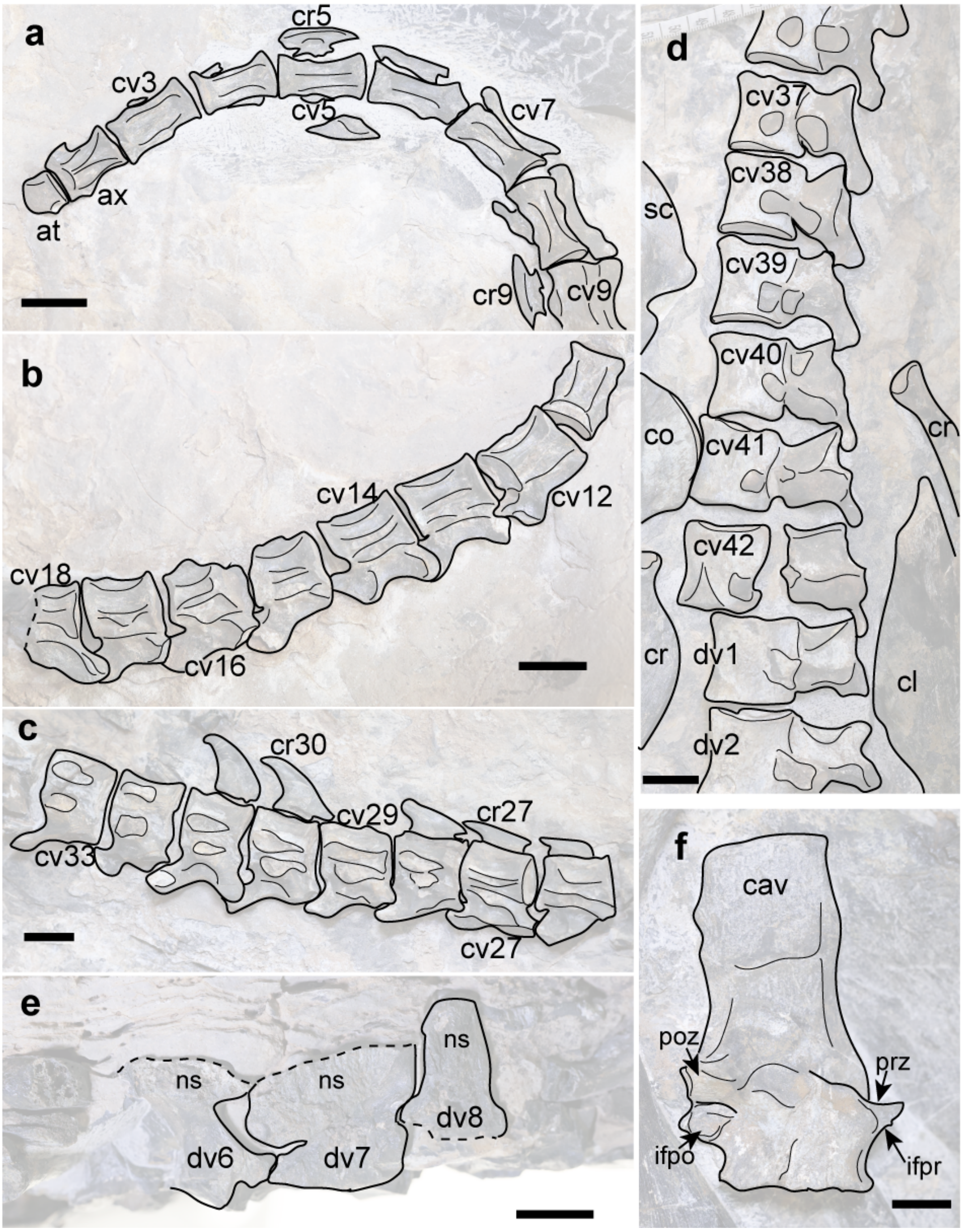

Figure S6. Interpretation on cervical and anterior dorsal vertebrae of the holotype of *Lijiangosaurus yongshengensis* (YSBB208). See explanation and abbreviations in Fig. S5. Scale bars are 1 cm in f and 2 cm in other images.

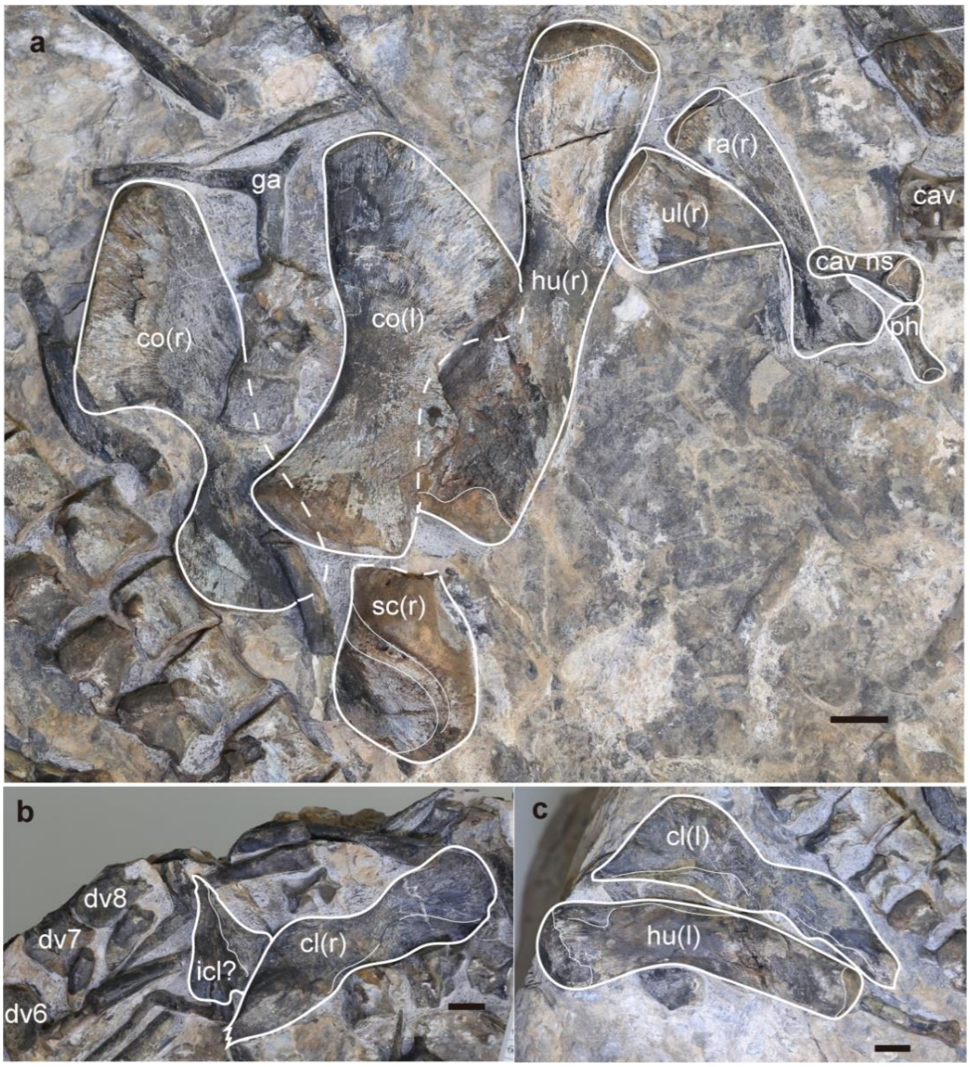

Figure S7. Pectoral girdle and forelimb elements of the holotype of *Lijiangosaurus yongshengensis* (YSBB208). Abbreviation: cav, caudal vertebra; cl, clavicle; co, coracoid; dv, dorsal vertebra; ga, gastralium; h, humerus; icl, interclavicle; (l), left; ns, neural spine; ph, phalanx; (r), right; ra, radius; sc, scapula; ul, ulna. Scale bars equal 2cm.

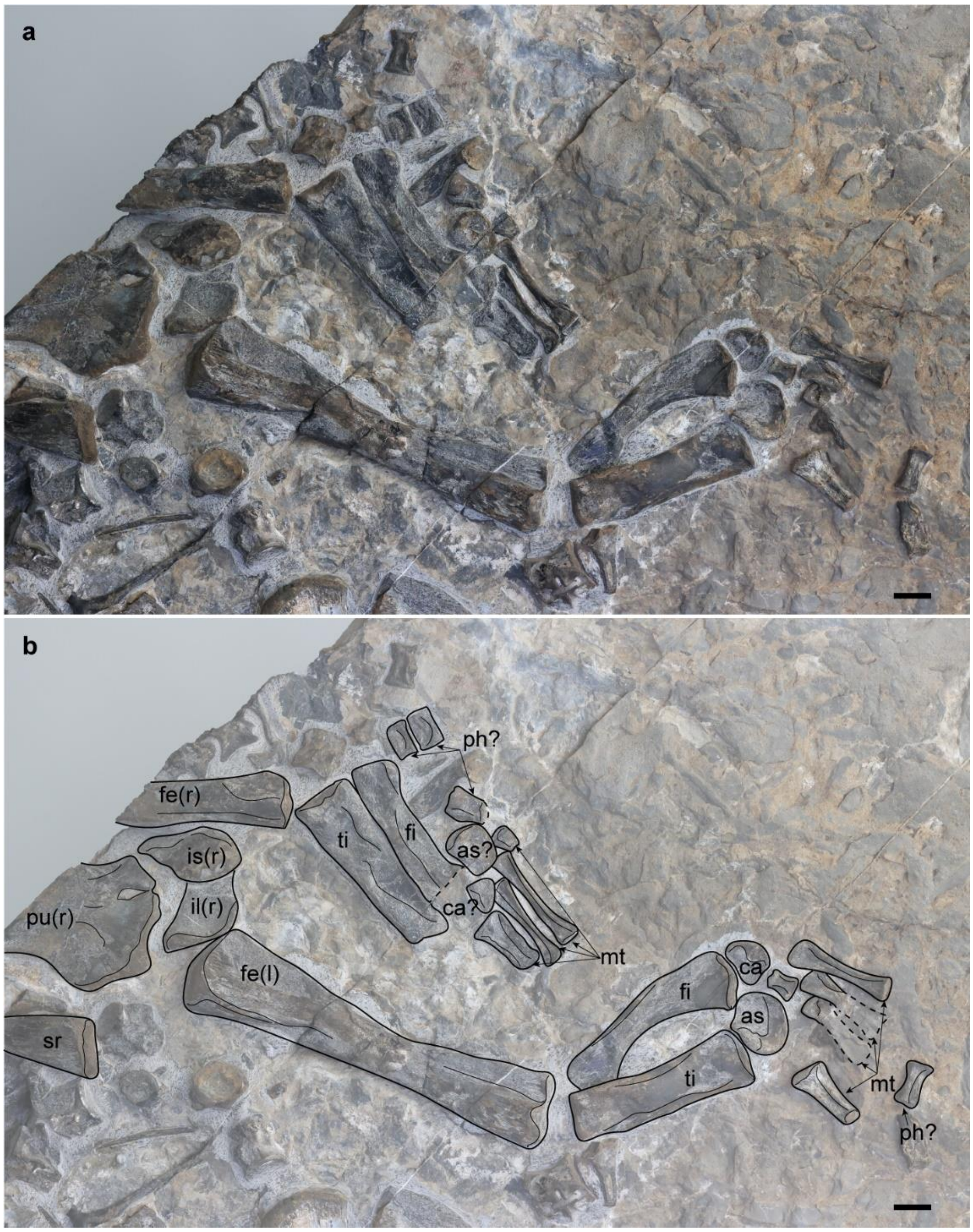

Figure S8. Pelvic girdle and hind limb elements of the holotype of *Lijiangosaurus yongshengensis* (YSBB208). The image (a) and the interpretation (b) of the preserved parts in situ. Abbreviation: as, astragalus; ca, calcaneum; fe, femur; fi, fibula; il, ilium; is, ischium; mt, metatarsal; ph, phalanx; pu, pubis; sr, sacral rib; ti, tibia. Scale bars equal 2 cm.

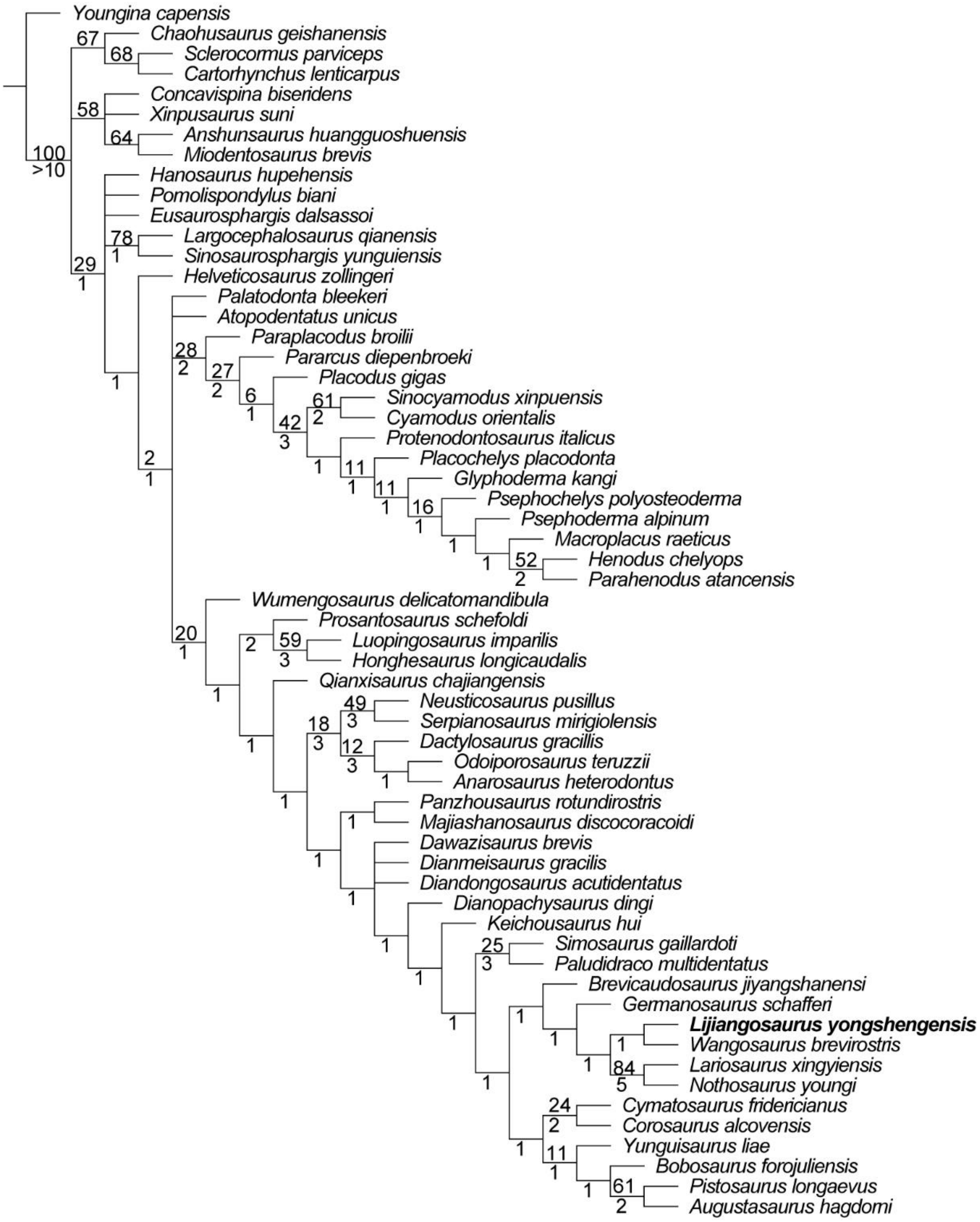

Figure S9. Strict consensus tree from the phylogenetic analysis using the dataset modified from Wang et al. (2022). Tree length is 783 steps, consistency index (CI) is 0.296, retention index (RI) is 0.708. Bootstrap values >10 and Bremer supports are labeled respectively above and under corresponding nodes.

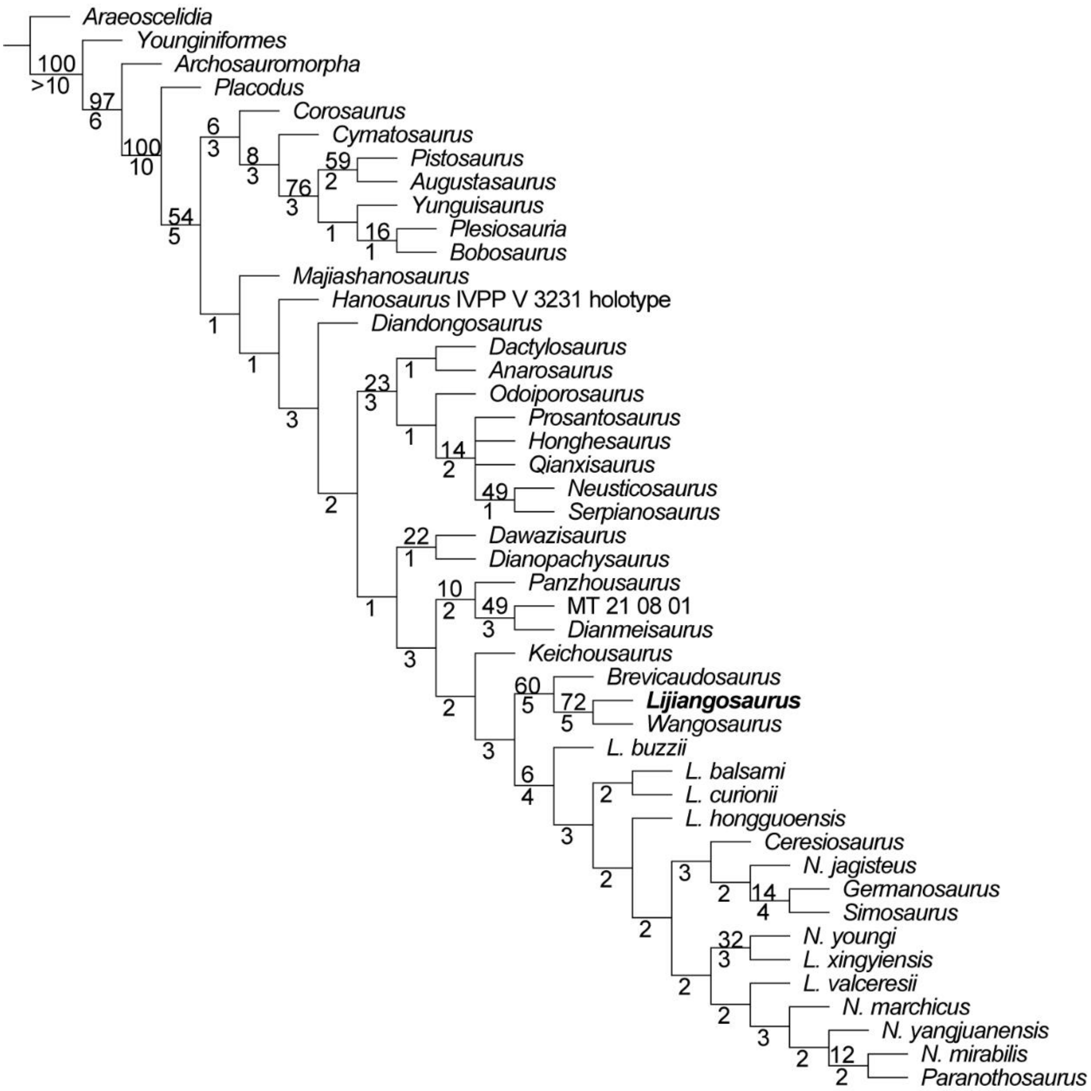

Figure S10. Strict consensus tree from the phylogenetic analysis using the dataset modified from Hu et al. (2024). Tree length is 868 steps, consistency index (CI) is 0.296, retention index (RI) is 0.604. Bootstrap values >10 and Bremer supports are labeled respectively above and under corresponding nodes.
