# Supplementary material for: Earliest long-necked sauropterygian Lijiangosaurus yongshengensis and plasticity of vertebral evolution in sauropterygian marine reptiles
Source: Commun Biol. 2025 Nov 11;8:1551. doi: 10.1038/s42003-025-08911-1 (PMC12606328; doi:10.1038/s42003-025-08911-1)
Supplement: Supplementary file 2 — Description of Additional Supplementary Materials [file 42003_2025_8911_MOESM2_ESM.pdf]

Description of Additional Supplementary Files

File name: Supplementary Data 1

Description: Character matrix modified from Wang et al. 2022.

File name: Supplementary Data 2

Description: Character matrix modified from Hu et al. 2024.
