# Supplementary material for: Earliest long-necked sauropterygian Lijiangosaurus yongshengensis and plasticity of vertebral evolution in sauropterygian marine reptiles
Source: Commun Biol. 2025 Nov 11;8:1551. doi: 10.1038/s42003-025-08911-1 (PMC12606328; doi:10.1038/s42003-025-08911-1)
Supplement: Supplementary file 5 — Reporting Summary [file 42003_2025_8911_MOESM5_ESM.pdf]

nature portfolio

Corresponding author(s): Wei Wang

Last updated by author(s): Sep 1, 2025

# Reporting Summary

Nature Portfolio wishes to improve the reproducibility of the work that we publish. This form provides structure for consistency and transparency in reporting. For further information on Nature Portfolio policies, see our Editorial Policies and the Editorial Policy Checklist.

## Statistics

For all statistical analyses, confirm that the following items are present in the figure legend, table legend, main text, or Methods section.

| n/a | Confirmed | |
|---|---|---|
| ☐ | ☒ | The exact sample size (*n*) for each experimental group/condition, given as a discrete number and unit of measurement |
| ☐ | ☒ | A statement on whether measurements were taken from distinct samples or whether the same sample was measured repeatedly |
| ☒ | ☐ | The statistical test(s) used AND whether they are one- or two-sided<br>*Only common tests should be described solely by name; describe more complex techniques in the Methods section.* |
| ☒ | ☐ | A description of all covariates tested |
| ☒ | ☐ | A description of any assumptions or corrections, such as tests of normality and adjustment for multiple comparisons |
| ☒ | ☐ | A full description of the statistical parameters including central tendency (e.g. means) or other basic estimates (e.g. regression coefficient) AND variation (e.g. standard deviation) or associated estimates of uncertainty (e.g. confidence intervals) |
| ☒ | ☐ | For null hypothesis testing, the test statistic (e.g. $F$, $t$, $r$) with confidence intervals, effect sizes, degrees of freedom and $P$ value noted<br>*Give P values as exact values whenever suitable.* |
| ☒ | ☐ | For Bayesian analysis, information on the choice of priors and Markov chain Monte Carlo settings |
| ☒ | ☐ | For hierarchical and complex designs, identification of the appropriate level for tests and full reporting of outcomes |
| ☒ | ☐ | Estimates of effect sizes (e.g. Cohen's *d*, Pearson's *r*), indicating how they were calculated |

*Our web collection on statistics for biologists contains articles on many of the points above.*

## Software and code

Policy information about availability of computer code

| | |
|---|---|
| Data collection | No software was used. |
| Data analysis | The character matrix was compiled in Mesquite v.2.75, and the parsimony analysis was conducted in TNT v. 1.6. |

For manuscripts utilizing custom algorithms or software that are central to the research but not yet described in published literature, software must be made available to editors and reviewers. We strongly encourage code deposition in a community repository (e.g. GitHub). See the Nature Portfolio guidelines for submitting code & software for further information.

## Data

Policy information about availability of data

All manuscripts must include a data availability statement. This statement should provide the following information, where applicable:

- Accession codes, unique identifiers, or web links for publicly available datasets
- A description of any restrictions on data availability
- For clinical datasets or third party data, please ensure that the statement adheres to our policy

The fossil specimen (YSBB208) described here is archived and available on request at the Yunnan Biantun Cultural Museum of Yongsheng in Lijiang, Yunnan Province, China. The morphologic data matrix and the measurement data are provided as Supplementary Data.

## Research involving human participants, their data, or biological material

Policy information about studies with human participants or human data. See also policy information about sex, gender (identity/presentation), and sexual orientation and race, ethnicity and racism.

| | |
|---|---|
| Reporting on sex and gender | n/a |
| Reporting on race, ethnicity, or other socially relevant groupings | n/a |
| Population characteristics | n/a |
| Recruitment | n/a |
| Ethics oversight | n/a |

Note that full information on the approval of the study protocol must also be provided in the manuscript.

# Field-specific reporting

Please select the one below that is the best fit for your research. If you are not sure, read the appropriate sections before making your selection.

☐ Life sciences ☐ Behavioural & social sciences ☒ Ecological, evolutionary & environmental sciences

For a reference copy of the document with all sections, see nature.com/documents/nr-reporting-summary-flat.pdf

# Life sciences study design

All studies must disclose on these points even when the disclosure is negative.

| | |
|---|---|
| Sample size | n/a |
| Data exclusions | n/a |
| Replication | n/a |
| Randomization | n/a |
| Blinding | n/a |

# Behavioural & social sciences study design

All studies must disclose on these points even when the disclosure is negative.

| | |
|---|---|
| Study description | n/a |
| Research sample | n/a |
| Sampling strategy | n/a |
| Data collection | n/a |
| Timing | n/a |
| Data exclusions | n/a |
| Non-participation | n/a |
| Randomization | n/a |

# Ecological, evolutionary & environmental sciences study design

All studies must disclose on these points even when the disclosure is negative.

| | |
|---|---|
| Study description | Our study described a Triassic fossil skeleton in comparison with multiple groups of marine reptiles. We constructed an updated data matrix on Triassic marine reptiles, conducted phylogenetic analyses, and erected a novel reptilian taxa. We discussed the cervical elongation and vertebral evolution in sauropterygian reptiles. |
| Research sample | This study is mainly based on the fossil skeleton YSBB208. Triassic marine reptile specimens used in this study are from many collections mainly in China and Europe. The phylogenetic analysis involved 68 species-level taxa and 181 morphological characters mostly based on direct observation. |
| Sampling strategy | The detailed description is mainly based on a single fossil specimen. The data is specifically selected to address the phylogenetic relationship and body plan variance of sauropterygiforms, and we sample the fossil material as broadly as possible with the preference of the complete adult skeletons. |
| Data collection | To avoid the potential bias, the leading author directly examined the true fossils in the collections in China and Europe as many as possible. All the data was observed, scored, and measured by the leading author with a consultation with other authors. |
| Timing and spatial scale | The core specimen was prepared by professional technicians under the supervision of the authors for one months. The data collected from the first-hand examination from December 2021 to May 2024. |
| Data exclusions | All the data for analyses was in Supplementary Information. No data were excluded. |
| Reproducibility | The main specimen YSBB208 and other fossil material used in this study are housed in permanent collections that are accessible. We provide the character matrix for phylogenetic analysis and the necessary description in the method. We believe that others can re-examine the specimens and use the data to reproduce our results. |
| Randomization | This study was mainly based on a single specimen. |
| Blinding | The description and other data are directly from the observation of fossils. |

Did the study involve field work? ☒ Yes ☐ No

## Field work, collection and transport

| | |
|---|---|
| Field conditions | In the fossil locality during the field work, the altitude is from 2000 m to 2500 m, the temperature is from 10 °C to 30 °C. |
| Location | Yongsheng, Lijiang, Yunnan Province, China. |
| Access & import/export | We investigated the fossil locality and prepare the specimen at the local museum. |
| Disturbance | The fossil has been prepared as much as possible to well expose anatomical features. |

# Reporting for specific materials, systems and methods

We require information from authors about some types of materials, experimental systems and methods used in many studies. Here, indicate whether each material, system or method listed is relevant to your study. If you are not sure if a list item applies to your research, read the appropriate section before selecting a response.

## Materials & experimental systems

| n/a | Involved in the study | |
|---|---|---|
| ☒ | ☐ | Antibodies |
| ☒ | ☐ | Eukaryotic cell lines |
| ☐ | ☒ | Palaeontology and archaeology |
| ☒ | ☐ | Animals and other organisms |
| ☒ | ☐ | Clinical data |
| ☒ | ☐ | Dual use research of concern |
| ☒ | ☐ | Plants |

## Methods

| n/a | Involved in the study | |
|---|---|---|
| ☒ | ☐ | ChIP-seq |
| ☒ | ☐ | Flow cytometry |
| ☒ | ☐ | MRI-based neuroimaging |

# Antibodies

| | |
|---|---|
| Antibodies used | n/a |
| Validation | n/a |

# Eukaryotic cell lines

Policy information about cell lines and Sex and Gender in Research

| | |
|---|---|
| Cell line source(s) | n/a |
| Authentication | n/a |
| Mycoplasma contamination | n/a |
| Commonly misidentified lines<br>(See ICLAC register) | n/a |

# Palaeontology and Archaeology

| | |
|---|---|
| Specimen provenance | The new specimen reported here was collected from the Beiya Formation in Yongsheng, Lijiang, Yunnan Province, China. It is prepared by professional technicians under the supervision of the authors. |
| Specimen deposition | The new specimen is permanently deposited in the native museum, Yunnan Biantun Cultural Museum of Yongsheng in Lijiang, Yunnan Province, with the specimen number YSBB208. |
| Dating methods | No new dates are provided here. The specimen was collected from and preserved in the limestone of the Beiya Formation with the available date from the literature. |

☒ Tick this box to confirm that the raw and calibrated dates are available in the paper or in Supplementary Information.

| | |
|---|---|
| Ethics oversight | Institute of Vertebrate Paleontology and Paleoanthropology, Chinese Academy of Sciences, Beijing, China. |

Note that full information on the approval of the study protocol must also be provided in the manuscript.

# Animals and other research organisms

Policy information about studies involving animals; ARRIVE guidelines recommended for reporting animal research, and Sex and Gender in Research

| | |
|---|---|
| Laboratory animals | n/a |
| Wild animals | n/a |
| Reporting on sex | n/a |
| Field-collected samples | n/a |
| Ethics oversight | n/a |

Note that full information on the approval of the study protocol must also be provided in the manuscript.

# Clinical data

Policy information about clinical studies

All manuscripts should comply with the ICMJE guidelines for publication of clinical research and a completed CONSORT checklist must be included with all submissions.

| | |
|---|---|
| Clinical trial registration | n/a |
| Study protocol | n/a |
| Data collection | n/a |
| Outcomes | n/a |

# Dual use research of concern

Policy information about dual use research of concern

## Hazards

Could the accidental, deliberate or reckless misuse of agents or technologies generated in the work, or the application of information presented in the manuscript, pose a threat to:

| No | Yes | |
|---|---|---|
| ☒ | ☐ | Public health |
| ☒ | ☐ | National security |
| ☒ | ☐ | Crops and/or livestock |
| ☒ | ☐ | Ecosystems |
| ☒ | ☐ | Any other significant area |

## Experiments of concern

Does the work involve any of these experiments of concern:

| No | Yes | |
|---|---|---|
| ☒ | ☐ | Demonstrate how to render a vaccine ineffective |
| ☒ | ☐ | Confer resistance to therapeutically useful antibiotics or antiviral agents |
| ☒ | ☐ | Enhance the virulence of a pathogen or render a nonpathogen virulent |
| ☒ | ☐ | Increase transmissibility of a pathogen |
| ☒ | ☐ | Alter the host range of a pathogen |
| ☒ | ☐ | Enable evasion of diagnostic/detection modalities |
| ☒ | ☐ | Enable the weaponization of a biological agent or toxin |
| ☒ | ☐ | Any other potentially harmful combination of experiments and agents |

# Plants

| | |
|---|---|
| Seed stocks | n/a |
| Novel plant genotypes | n/a |
| Authentication | n/a |

# ChIP-seq

## Data deposition

☐ Confirm that both raw and final processed data have been deposited in a public database such as GEO.

☐ Confirm that you have deposited or provided access to graph files (e.g. BED files) for the called peaks.

| | |
|---|---|
| Data access links<br>*May remain private before publication.* | n/a |
| Files in database submission | n/a |
| Genome browser session<br>(e.g. UCSC) | n/a |

## Methodology

| | |
|---|---|
| Replicates | n/a |

| | |
|---|---|
| Sequencing depth | n/a |
| Antibodies | n/a |
| Peak calling parameters | n/a |
| Data quality | n/a |
| Software | n/a |

# Flow Cytometry

## Plots

Confirm that:

☒ The axis labels state the marker and fluorochrome used (e.g. CD4-FITC).

☒ The axis scales are clearly visible. Include numbers along axes only for bottom left plot of group (a 'group' is an analysis of identical markers).

☒ All plots are contour plots with outliers or pseudocolor plots.

☒ A numerical value for number of cells or percentage (with statistics) is provided.

## Methodology

| | |
|---|---|
| Sample preparation | n/a |
| Instrument | n/a |
| Software | n/a |
| Cell population abundance | n/a |
| Gating strategy | n/a |

☒ Tick this box to confirm that a figure exemplifying the gating strategy is provided in the Supplementary Information.

# Magnetic resonance imaging

## Experimental design

| | |
|---|---|
| Design type | n/a |
| Design specifications | n/a |
| Behavioral performance measures | n/a |

## Acquisition

| | |
|---|---|
| Imaging type(s) | n/a |
| Field strength | n/a |
| Sequence & imaging parameters | n/a |
| Area of acquisition | n/a |

Diffusion MRI ☐ Used ☒ Not used

## Preprocessing

| | |
|---|---|
| Preprocessing software | n/a |
| Normalization | n/a |
| Normalization template | n/a |

Noise and artifact removal: n/a

Volume censoring: n/a

## Statistical modeling & inference

Model type and settings: n/a

Effect(s) tested: n/a

Specify type of analysis: ☐ Whole brain ☐ ROI-based ☐ Both

Statistic type for inference (See Eklund et al. 2016): n/a

Correction: n/a

## Models & analysis

| n/a | Involved in the study |
| --- | --- |
| ☒ | ☐ Functional and/or effective connectivity |
| ☒ | ☐ Graph analysis |
| ☒ | ☐ Multivariate modeling or predictive analysis |
